# Association between *Moraxella* keratitis and advanced glycation end products

**DOI:** 10.1038/s41598-024-58659-7

**Published:** 2024-04-05

**Authors:** Hidenori Inoue, Koji Toriyama, Naoko Takahira, Shinobu Murakami, Hitoshi Miyamoto, Takashi Suzuki, Atsushi Shiraishi

**Affiliations:** 1 Department of Ophthalmology, Ehime University Graduate School of Medicine, Shitsukawa, Toon, Ehime 791-0295 Japan; 2 Clinical Laboratory Division, Ehime University Hospital, Shitsukawa, Toon, Ehime 791-0295 Japan; 3 Department of Ophthalmology, Toho University Graduate School of Medicine, 6-11-1, Omori-nishi, Ota-ku, Tokyo, 143-8541 Japan

**Keywords:** Corneal diseases, Bacterial infection, Diabetes

## Abstract

Diabetes mellitus is recognized as a major predisposing factor for *Moraxella* keratitis. However, how diabetes mellitus contributes to *Moraxella* keratitis remains unclear. In this study, we examined *Moraxella* keratitis; based on the findings, we investigated the impact of advanced glycation end products (AGEs) deposition in the cornea of individuals with diabetic mellitus on the adhesion of *Moraxella* isolates to the cornea. A retrospective analysis of 27 culture-proven cases of *Moraxella* keratitis at Ehime University Hospital (March 2006 to February 2022) was performed. *Moraxella* isolates were identified using matrix-assisted laser desorption ionization time-of-flight mass spectrometry. Among the patients, 30.4% had diabetes mellitus and 22.2% had the predominant ocular condition of using steroid eye drops. The species identified were *Moraxella nonliquefaciens* in 59.3% and *Moraxella lacunata* in 40.7% of patients. To investigate the underlying mechanisms, we assessed the effects of *M. nonliquefaciens* adherence to simian virus 40-immortalized human corneal epithelial cells (HCECs) with or without AGEs. The results demonstrated the number of *M. nonliquefaciens* adhering to HCECs was significantly increased by adding AGEs compared with that in controls (*p* < 0.01). Furthermore, in the corneas of streptozotocin-induced diabetic C57BL/6 mice treated with or without pyridoxamine, an AGE inhibitor, the number of *M. nonliquefaciens* adhering to the corneas of diabetic mice was significantly reduced by pyridoxamine treatment (*p* < 0.05). In conclusion, the development of *Moraxella* keratitis may be significantly influenced by the deposition of AGEs on the corneal epithelium of patients with diabetes mellitus.

## Introduction

*Moraxella* species were isolated from a patient with conjunctivitis by Morax in 1896, and the following year, Axenfeld isolated them alone^1^ They present as Gram-negative, twin-short bacilli and are common in the mucous membranes of the human oral cavity, nasal cavity, and pharynx^2,3^ *Moraxella* species cause otitis media, sinusitis, laryngitis, pneumonia, and meningitis, and *Moraxella catarrhalis* is the primary causative agent of respiratory tract infections^2,4–7^ In the field of ophthalmology, *Moraxella* species are etiological agents of various infectious diseases, such as conjunctivitis, keratitis, blepharitis, and endophthalmitis, and various *Moraxella* species, including *M. catarrhalis*, have been reported as causative organisms^8–20^.

*Moraxella* species are the second most significant Gram-negative rods of infectious keratitis, following *Pseudomonas aeruginosa*^21–23^ Diabetes mellitus has been previously reported as a major systemic predisposing factor for *Moraxella* keratitis^9,11,12,14–16,18–20^ Diabetes mellitus affects the tear fluid, such as decreased volume, increased glucose concentration, intracorneal nerve abnormalities, and metabolic abnormalities in the corneal epithelium, resulting in diabetic keratopathy^24^ Metabolic abnormalities cause an increase in the concentrations of aldose reductase and deposition of advanced glycation end products (AGEs) in diabetic keratopathy^25,26^ The Maillard process, which is the nonenzymatic glycation of proteins, produces AGEs as byproducts^27^ AGEs accumulate throughout the body and cause various diseases, including cardiovascular and renal diseases^28^ The accumulation process of AGEs is caused by cell metabolic dysfunction and changes in various macromolecule structures, which impact the physiological activities of various eye components^29–33^ Furthermore, the formation of AGE is closely related to aging eye pathologies, such as cataracts and diabetic retinopathy^29–33^ AGE deposition in corneal layers, including epithelium, Bowman's layer, and endothelium, has been shown in monkeys, mice, and humans with diabetes mellitus^29,34,35^ According to Kaji et al.^29^, AGE deposition on laminin in the corneal epithelial basement membrane results in impaired laminin extension and corneal epithelial defects. Shi et al.^36^ found that AGEs induce apoptosis of corneal epithelial cells. Therefore, AGEs have various effects on the cornea and are associated with infection. Ozer et al.^37^ reported that AGEs play a role in bacterial adhesion to the bladder epithelium in urinary tract infections. To the best of our knowledge, a link between AGEs and ocular infections has never been reported. Bacterial adhesion to biological surfaces is essential for the development of infectious diseases^38,39^. We hypothesize that increased adhesion of *Moraxella* to the corneal epithelium in patients with diabetes mellitus may be a reason why diabetes mellitus is a major systemic predisposing factor for *Moraxella* keratitis. In this study, we retrospectively reviewed the species and predisposing factors for *Moraxella* keratitis at our institution and focused on AGE deposition on the cornea of patients with diabetes mellitus. We examined changes in the ability of *Moraxella nonliquefaciens*, which is considered a major species associated with *Moraxella* keratitis, to adhere to the corneal epithelium in the presence or absence of AGEs.

## Results

### Clinical features of *Moraxella* keratitis

Among the 27 patients included in this study, whose ages ranged from 28 to 94 years, 16 were male and 11 were female. Using matrix-assisted laser desorption ionization time-of-flight mass spectrometry (MALDI-TOF MS), we successfully identified each isolate from the specimens of *Moraxella* keratitis. This led to a distinction between two prevalent *Moraxella* species: *M. nonliquefaciens* in 59.3% (16 isolates) and *Moraxella lacunata* in 40.7% (11 isolates) of cases. The majority of patients (77.8%) significantly presented with ocular-predisposing factors, as detailed in Table 1. Notably, 22.2% of these patients used steroid eye drops, and common ocular conditions like bullous keratopathy, dry eye, penetrating keratoplasty, lagophthalmos, and trauma were observed in 7.4% of the cases. Additionally, our investigation of systemic predisposing factors in 23 out of the 27 patients revealed that 60.9% of them had relevant systemic risk factors that included diabetes mellitus in 30.4%, use of anticancer drugs in 26.1%, and Sjögren’s syndrome in 4.3% of the cases.

**Table 1:** Predisposing factors for *Moraxella* keratitis.

*n* (%)	
Ocular predisposing factors	*n* = 27
Steroid eye drops	6 (22.2)
Bullous keratopathy	2 (7.4)
Dry eye	2 (7.4)
Penetrating keratoplasty	2 (7.4)
Lagophthalmos	2 (7.4)
Trauma	2 (7.4)
Others	5 (18.5)
Total	21 (77.8)
Systemic predisposing factors	*n* = 23
Diabetes mellitus	7 (30.4)
Anticancer drugs	6 (26.1)
Sjögren syndrome	1 (4.3)
Total	14 (60.9)

### In Vitro adhesion of *M. nonliquefaciens* to human corneal epithelial cells immortalized by simian virus 40

**Figure 1:**
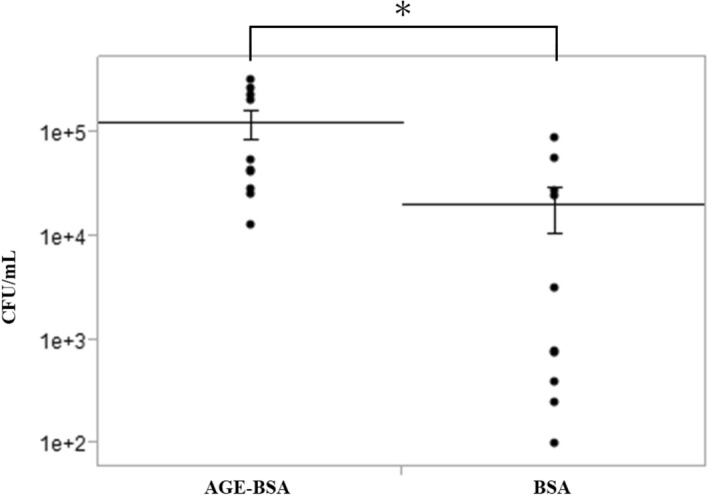
Effects of AGEs on the ability of *Moraxella nonliquefaciens* to adhere to HCECs. The number of adherent *M. nonliquefaciens* was significantly higher in HCECs with AGE-BSA. Two-tailed unpaired *t*-test, *p < 0.01. The mean ± *SEM* of CFU/mL for each group is shown by the central horizontal lines bisected by error bars (*n* = 10 wells/group). AGE, advanced glycation end product; BSA, bovine serum albumin; HCEC, human corneal epithelial cells; SEM, standard error of the mean.

The effect of AGEs on the adhesion of *M. nonliquefaciens* to human corneal epithelial cells (HCECs) immortalized by SV 40 was examined. The number of adherent *M. nonliquefaciens* was compared between HCECs supplemented with 100 µg/mL advanced glycation end product–bovine serum albumin (AGE-BSA) and those supplemented with bovine serum albumin (BSA) alone. The number of adherent *M. nonliquefaciens* was significantly higher in HCECs supplemented with 100 µg/mL AGE-BSA (*p* < 0.05 for the two-tailed unpaired t-test; Fig. 1).

### Immunohistochemical localization of AGEs in the mouse cornea

**Figure 2:**
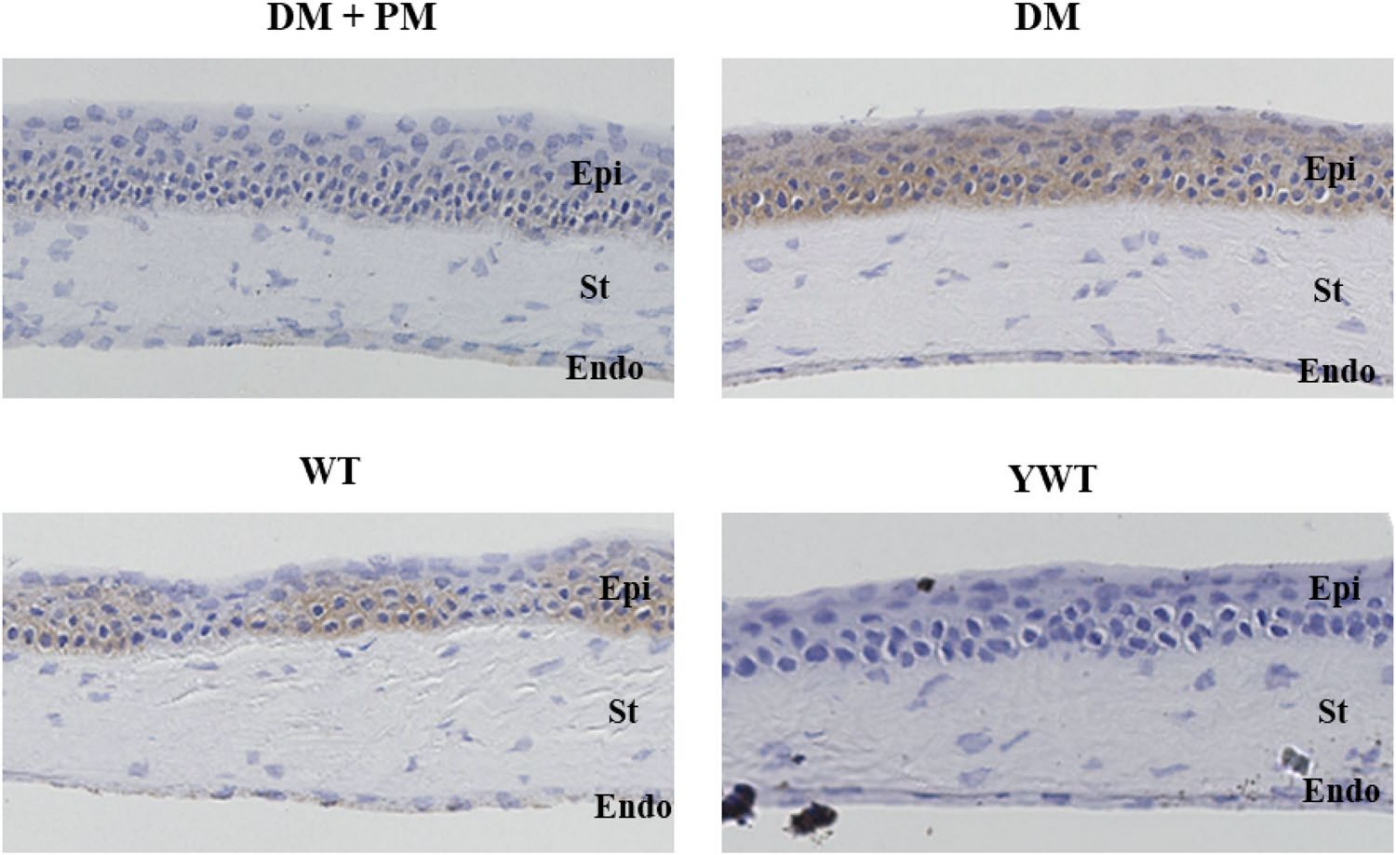
AGE immunoreactivity in the corneas of diabetic mice that consumed PM, diabetic mice, age-matched wild-type mice, and young wild-type mice. Diabetic mice treated with PM showed almost no AGE reactivity in the cornea. Diabetic mice showed AGE reactivity diffusely in all layers of the corneal epithelium and endothelial cells. Age-matched wild-type mice showed moderate AGE reactivity in the deep corneal epithelium, basal layer of the corneal epithelium, and endothelial cells. Young wild-type mice showed no AGE reactivity. AGE, advanced glycation end product; DM, diabetes mellitus; Endo, endothelium; Epi, epithelium; PM, pyridoxamine; St, stroma; WT, age-matched wild-type; YWT, young wild-type.

The immunohistochemistry (IHC) results showed AGE immunoreactivity in the corneas of diabetic mice that consumed pyridoxamine, diabetic mice, age-matched wild-type mice, and young wild-type mice (Fig. 2). In diabetic mice, AGE reactivity was diffusely observed in all layers of the corneal epithelium and endothelial cells. In contrast, in diabetic mice that consumed pyridoxamine, marginal AGE reactivity was observed in the basal layer of the corneal epithelium and endothelial cells. In age-matched wild-type mice, AGE reactivity was observed in the deep corneal epithelium, basal layer of the corneal epithelium, and endothelial cells. Young wild-type mice showed no AGE reactivity.

### Ex Vivo adhesion of *M. nonliquefaciens* to the mouse cornea

**Figure 3:**
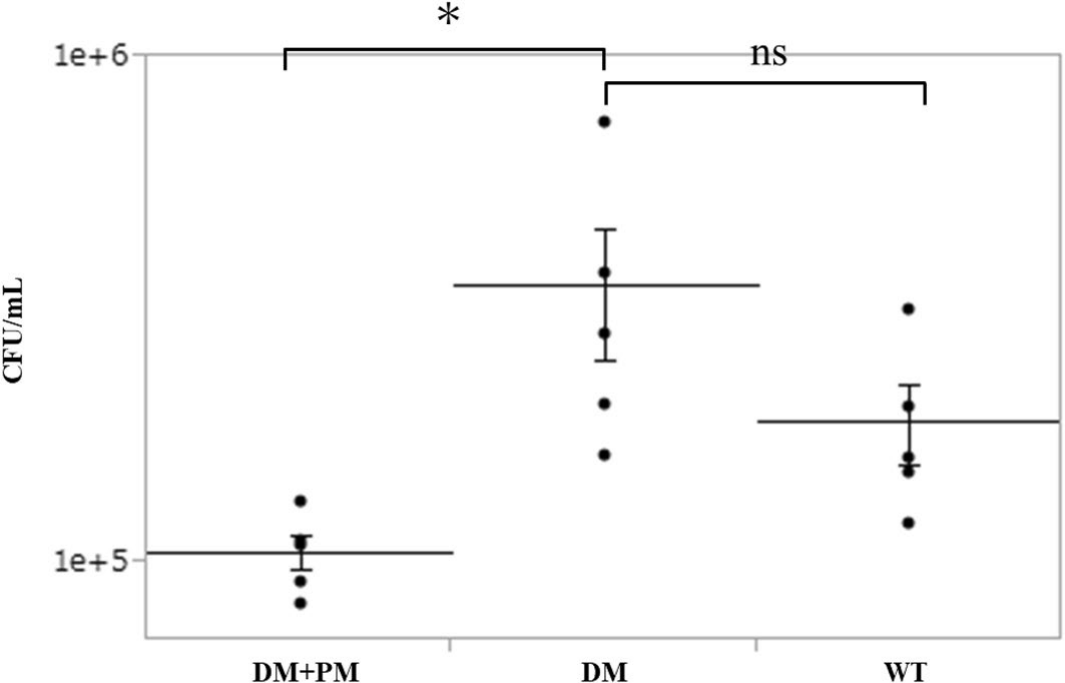
The effect of AGEs on the ability of *Moraxella nonliquefaciens* to adhere to the mouse corneas. The number of adherent *M. nonliquefaciens* was significantly higher in the corneas of diabetic mice than those with diabetes mellitus treated with PM. Two-tailed unpaired *t*-test, *p < 0.05. There was no significant difference in the number of adhesions between diabetic mice and age-matched wild-type mice. Two-tailed unpaired *t*-test, *p* = 0.09. The mean ± *SEM* of CFU/mL for each group is shown by the central horizontal lines bisected by the error bars (*n* = 5 mice/group). AGE, advanced glycation end product; DM, diabetes mellitus; PM, pyridoxamine; WT, age-matched wild-type; SEM, standard error of the mean.

**Figure 4:**
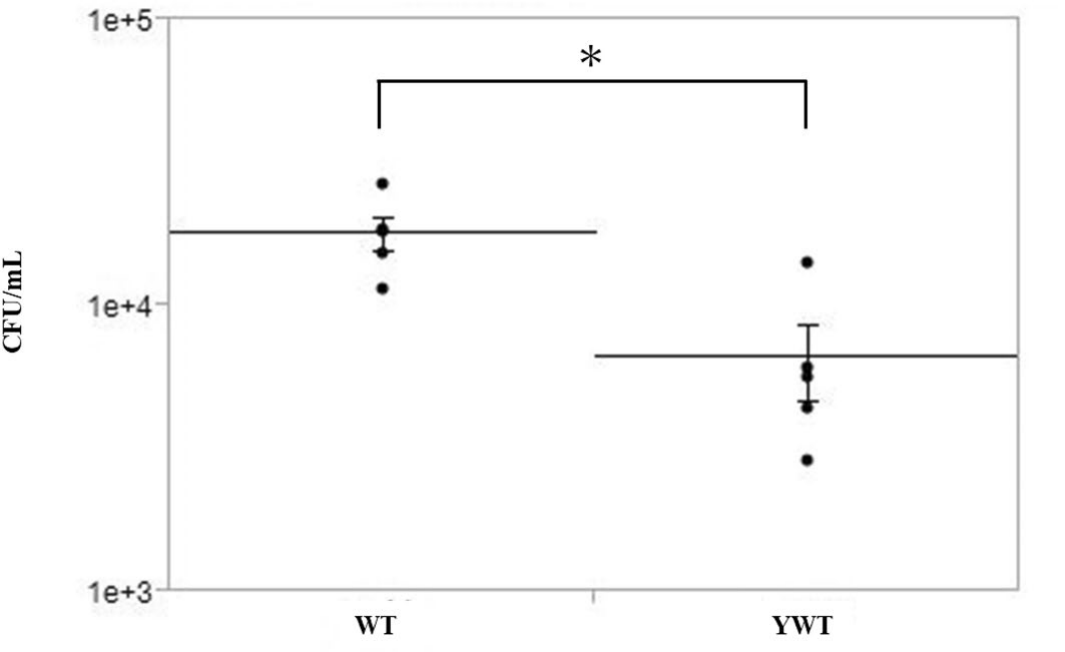
The effect of AGEs on the ability of *Moraxella nonliquefaciens* to adhere to the mouse corneas. The number of adherent *M. nonliquefaciens* was significantly higher in the corneas of age-matched wild-type mice than in those of young wild-type mice. Two-tailed unpaired *t*-test, *p < 0.05. The mean ± *SEM* of CFU/mL for each group is shown by the central horizontal lines bisected by the error bars (*n* = 5 mice/group). AGE, advanced glycation end product; WT, age-matched wild-type; YWT, young wild-type; SEM, standard error of the mean.

The number of adherent *M. nonliquefaciens* was significantly higher in the corneas of diabetic mice than in those of diabetic mice that consumed pyridoxamine (*p* < 0.05 for two-tailed unpaired t-test; Fig. 3). In contrast, no significant difference in the number of adherent *M. nonliquefaciens* was observed between the corneas of diabetic mice and those of age-matched wild-type mice, although the number of adhesions tended to be higher in diabetic mice. The number of adherent *M. nonliquefaciens* was significantly higher in the corneas of age-matched wild-type mice than in those of young wild-type mice (*p* < 0.05, two-tailed unpaired t-test; Fig. 4).

## Discussion

Clinical features of *Moraxella* keratitis were reviewed retrospectively, demonstrating *M. nonliquefaciens* as the main etiological agent of corneal infection associated with diabetes mellitus. The identification of *Moraxella* species is difficult in laboratories because of their similar biochemical properties^12^. Recently, a quick and accurate approach for identifying microbes has emerged: MALDI-TOF-MS^40,41^. Thus, identifying the species of *Moraxella* responsible for keratitis is now possible. In this study, only two species, *M. nonliquefaciens and M. lacunata,* were identified in keratitis. These species have been reported to be major causative species of *Moraxella* keratitis in previous reports^12,16–19^. In support of our results, LaCroce et al. reported that *M. nonliquefaciens* was the most frequently identified organism in cases of *Moraxella* keratitis (80.5%)^17^. Thus, *M. nonliquefaciens* is considered a major species that causes *Moraxella* keratitis. Moreover, these two species do not reside on the ocular surface; rather, they are endemic in the oral cavity and upper respiratory tract and can be transient bacteria on the ocular surface^42^.

Diabetes mellitus was previously reported as a major predisposing factor for *Moraxella* keratitis, with frequency ranging from 7.3% to 23.3%^9,11,12,14–16,18–20^. Similar to other studies, 30.4% of the patients in this study had diabetes mellitus.

Adhesion is essential for bacteria to develop an infection, and the same is true for infectious keratitis^38,39^. Reichert et al. reported that the difference in pathogenicity of bacteria in infectious keratitis is related to their ability to adhere to the corneal epithelium^43^. To the best of our knowledge, the ability of *Moraxella* to adhere to the corneal epithelium has not yet been evaluated. *Pseudomonas aeruginosa*, a Gram-negative rod bacterium, has type 4 pili required for adhesion to corneal epithelial cells^44^. *Moraxella* species also have type 4 pili^45,46^. Ozer et al. reported that the binding of AGEs to the pili of uropathogenic *Escherichia coli* (UPEC) increased the adhesion of UPEC to the urothelium^37^. We hypothesized that similar changes might be occurring in the cornea. The adhesion of *Moraxella* to the corneal epithelium may be increased by binding the pili of *Moraxella* to AGEs in the corneal epithelium deposited by diabetes mellitus. To clarify this hypothesis, we examined the adhesion of *M. nonliquefaciens* to the corneal epithelium in vitro using HCECs and ex vivo using the corneas of mice.

The number of adherent *M. nonliquefaciens* to HCECs was significantly increased by the addition of AGEs to HCECs (Fig. 1). Ozer et al. reported that in vitro binding of type 1 pili to several AGEs enhances the adhesion of UPEC to the urothelium, and Fim H, an adhesin, may be involved in type 1 pili binding to AGEs^37^. Type 1 pili are classified as chaperone-usher pili, and Gram-negative rods universally express chaperone-usher pili on their outer surfaces^47,48^. Pili play an essential role in the adhesion of bacteria to host tissues, particularly the adhesin at the tip of the pilis^49^. Thus, we hypothesized that the adhesion of *M. nonliquefaciens* to the corneal epithelium is facilitated by binding of these pilis to AGEs deposited in the corneal epithelium. Further studies are required to determine whether AGEs bind to the pili of *M. nonliquefaciens.*

To confirm the possibility that AGEs enhance the ability of *M. nonliquefaciens* to adhere to the corneal epithelium, we induced diabetes mellitus in mice in which AGEs were deposited in the cornea and conducted ex vivo experiments using the corneas of these mice. In this study, IHC results showed that AGEs were highly deposited in the entire corneal epithelium, epithelial basement membrane, and endothelial cells in diabetic mice (Fig. 2). This is consistent with previous studies that used mice and monkeys with diabetes mellitus^34,35^. The high blood glucose level in diabetic mice is believed to cause a Maillard reaction due to excess sugar, resulting in the deposition of high levels of AGEs^50^. AGEs have also been reported to accumulate with aging^51,52^. Our results support these previous reports, as moderate AGE deposition was observed in the cornea of age-matched wild-type mice. In this study, pyridoxamine, which is a natural type of vitamin B, was used as an AGE inhibitor. Pyridoxamine inhibits the formation of AGEs from Amadori intermediates by blocking the Maillard reaction^53^. Pyridoxamine inhibits the deposition of AGEs in various animal studies^35,37,54^ and mouse cornea^35^. In this study, we confirmed that pyridoxamine suppressed the deposition of AGEs in the cornea of mice with streptozotocin (STZ) -induced diabetes mellitus (Fig. 2). Ex vivo examination demonstrated that *M. nonliquefaciens* adhered more readily to the corneal epithelium in the following order: diabetic mice, age-matched wild-type mice, and diabetic mice that consumed pyridoxamine. Furthermore, the number of bacteria adhering to the cornea was significantly higher in diabetic mice than those that consumed pyridoxamine (Fig. 3). These results are consistent with the degree of AGE deposition in each mouse cornea, as demonstrated by IHC examination, confirming that the number of adherent *M. nonliquefaciens* in the cornea increases with AGE deposition. This suggests that AGE deposition in the cornea affects the ability of *M. nonliquefaciens* to adhere to the corneal epithelium. In this study, diabetic mice and age-matched wild-type mice showed no significant difference in the number of *Moraxella* adhesions to the cornea, although that number tended to be higher in diabetic mice. The mice in this study were 24 weeks old, which may affect AGE deposition in the cornea and bacterial adhesion. IHC of corneas removed from young wild-type mice (8-week-old) showed no AGE immunoreactivity (Fig. 2). This suggests that aging also induces the accumulation of AGEs in the cornea. An ex vivo examination of age-matched and young wild-type mice showed a significantly higher number of *M. nonliquefaciens* adhesions in the corneas of age-matched wild-type mice (Fig. 4). This finding is consistence with the results of IHC. *Moraxella* keratitis occurs more frequently in the elderly^11,22^, possibly related to AGE deposition in the cornea due to aging.

Some limitations of this study should be acknowledged. First, how *M. nonliquefaciens* adheres to AGEs in the corneal epithelium remains uncertain. Second, we have not investigated whether changes in the cornea with diabetes mellitus other than AGE deposition affect the ability of *M. nonliquefaciens* to adhere to the corneal epithelium. Further studies are necessary to clarify the relationship between *M. nonliquefaciens* and diabetes mellitus.

In future, we will focus on elucidating the molecular interactions between AGEs and adhesins of the pili present in *M. nonliquefaciens* to improve our understanding of the pathogenesis of *Moraxella* keratitis, especially in patients with diabetes. Furthermore, the exploration of therapeutic strategies for inhibiting *Moraxella*-AGE binding offers novel promising approaches for treatment. Finally, the study of similar bacterial-adhesion mechanisms in other ocular diseases may broaden the scope of this research.

In conclusion, this study offers a fresh understanding of the effects of AGE deposition on bacterial adhesion to the cornea. We have shown in vitro and ex vivo that AGE deposition in the cornea increases the adhesion of *M. nonliquefaciens* to the cornea. The results suggest that AGE deposition in the cornea of patients with diabetes mellitus is a predisposing factor for developing *Moraxella* keratitis.

## Methods

### Ethics

The Institutional Review Board (IRB) of Ehime University approved this study in accordance with the principles of the Declaration of Helsinki (approval number 1503007). Written informed consent was obtained from all patients. The ARRIVE guidelines were followed in performing all methods used in animal tests, which were approved by the Institutional Animal Care and Use Committee of Ehime University (approval number 05HA84-1). Additionally, all the methods were carried out in accordance with relevant Institutional guidelines and regulations.

### Patients and clinical features

Clinical and microbiological records of 27 culture-proven *Moraxella* keratitis cases reported at Ehime University Hospital from March 2006 to February 2022 were retrospectively analyzed in addition to the species and predisposing factors for *Moraxella* keratitis. The organisms were identified by Gram-stained smears and corneal scrape culture on sheep blood agar at 37 °C and 5% CO_2_. On a Bruker MALDI Biotyper (Bruker Daltonics, Billerica, MA), MALDI-TOF MS was performed to identify *Moraxella* species. The identification criteria were a score of ≥ 2.0 for species-level identification.

### Cell culture

HCECs immortalized by SV40 were purchased from the RIKEN BioResource Research Center (Ibaraki, Japan) and used. SV40 HCECs were cultured in a supplemental hormonal epithelial medium (SHEM) comprising Dulbecco’s Modified Eagle Medium/F-12 (1:1) from Life Technologies in Grand Island, New York, with 1% antibiotic–antimycotic combination, 0.5 μg/mL hydrocortisone, 0.1 μg/mL cholera toxin, and 0.5% dimethyl sulfoxide in 37 °C humid atmosphere with 5% CO_2_. Before testing, 24-well plates of HCECs (2.0 × 10^4^ cells/well) were grown to 95% confluence. The concentration of HCECs was determined using an automated cell counter (Countess III automated cell counter; Thermo Fisher Scientific, Waltham, Massachusettes).

### Bacterial strain

**Figure 5:**
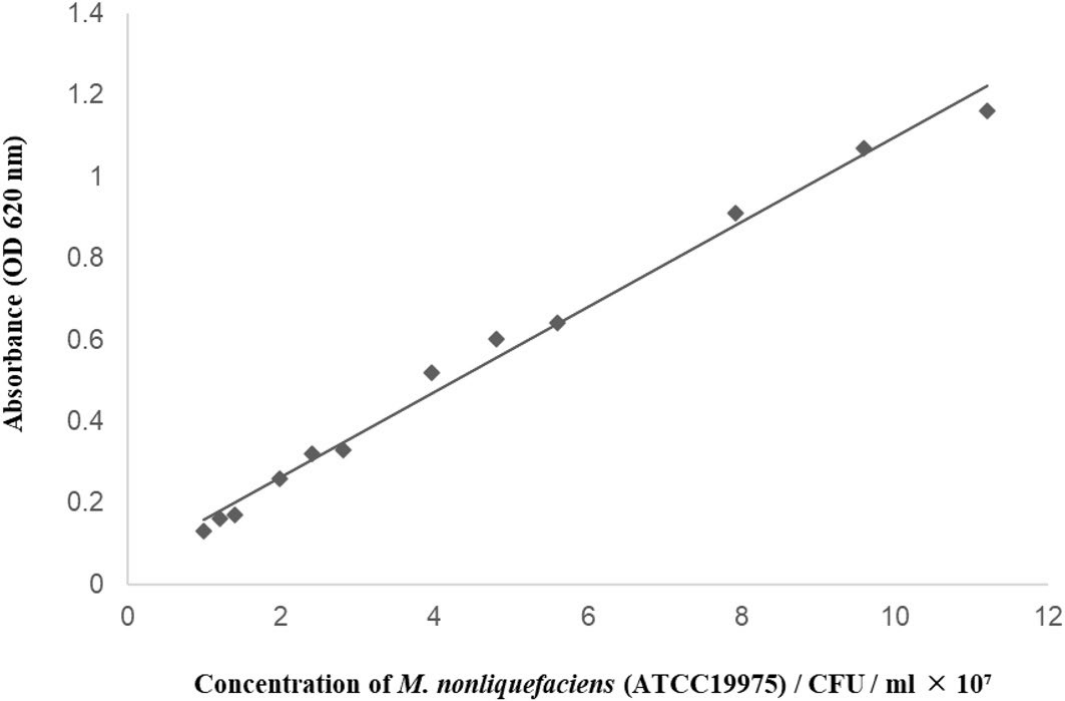
Calibration curve of *Moraxella nonliquefaciens* (ATCC19975). y = 0.1043x + 0.0546, R^2^ = 0.9914.

A standard strain of *M. nonliquefaciens* ATCC (American Type Culture Collection) 19,975 was used in each experiment. The strain was raised for 24 h at 37 °C and 5% CO_2_ in brain–heart infusion broth (Becton Dickinson, Sunnyvale, CA). The bacterial solution was then diluted with phosphate-buffered saline (PBS) to the appropriate concentration. A calibration curve for *M. nonliquefaciens* (ATCC19975) was constructed to adjust the concentration (Fig. 5). To establish the calibration curve, the turbidity of the bacterial solution was measured using an absorbance meter (CO8000 Biowave Personal Cell Density Meter; Biochrom, Cambridge, UK), and solution was plated onto blood agar for counting the number of colonies.

### Animals

In this study, 7-week-old male C57BL/6 mice were purchased from CLEA Japan Inc. (Tokyo, Japan). STZ (Sigma-Aldrich, St. Louis, Missouri) (200 mg/kg) was intraperitoneally administered once in 8-week-old male C57BL/6 mice to induce diabetes mellitus in mice^55^. Diabetic mice with blood glucose levels > 300 mg/dL were used in the experiments. The AGE inhibitor pyridoxamine dihydrochloride (Tokyo Chemical Industry, Tokyo, Japan) in 1 g/L of water was used to suppress AGE deposition in the cornea of diabetic mice. Male C57BL/6 mice began consuming this water 1 week before the intraperitoneal administration of STZ^37^. We raised these mice until 24 weeks of age and used them for experiments. Diabetic mice (24-week-old), diabetic mice that consumed pyridoxamine (24-week-old), age-matched wild-type mice (24-week-old), and young wild-type mice (8-week-old) were deeply anesthetized with a cocktail of medetomidine hydrochloride (1.0 mg), midazolam (5.0 mg/mL), and butorphanol (5.0 mg/mL). After anesthesia induction, the mice were euthanized by de-bleeding from the axillary artery, and only the cornea was removed and used in the experiments.

### In Vitro adhesion of *M. nonliquefaciens* to HCECs

An adhesion assay was performed essentially as previously described^56,57^. The concentrations of AGEs and bacteria were determined based on previous reports^36,58^. Advanced glycation end product–bovine serum albumin (AGE-BSA) (Cayman Chemical, Ann Arbor, MI) was added to 1 mL of SHEM to achieve a concentration of 100 μg/mL in 10 wells, and 1 mL of SHEM containing BSA was added to another 10 wells in a 24-well plate applied to HCECs (2.0 × 10^4^ cells/well). The plate was incubated at 37 °C in a humid atmosphere with 5% CO_2_ for 12 h. After incubation, we removed the old medium from each well and added 1 mL of SHEM containing 30 μL of the bacterial solution. The bacterial solution was adjusted to 1.0 × 10^8^ CFU/mL using the calibration curve. To encourage the bacteria to contact the HCECs, the 24-well plates were centrifuged at 100 g for 5 min. They were then incubated at 37 °C with 5% CO_2_ for 1 h. After incubation, the supernatants were discarded and the HCECs were washed with warm PBS. The HCECs were stripped by immersing them in 150 μL of 0.05% Trypsin–EDTA (Life Technologies, Grand Island, NY) for 5 min, diluted with PBS, and applied to a sheep blood agar. The agar was incubated for 24 h at 37 °C in a humid atmosphere with 5% CO_2_, and the number of colonies on the agar was counted manually. This test was performed three times.

### Immunohistochemistry

The corneas removed from diabetic mice, diabetic mice that consumed pyridoxamine, age-matched wild-type mice, and young wild-type mice were fixed in 4% paraformaldehyde for 1 h. Subsequently, 5 µm-thick sections of the corneas were cut. The sections were graded, ethanol-dehydrated, and deparaffinized using xylene. Then, the sections were deparaffinized with xylene, dehydrated with graded ethanol, and immersed in PBS containing 0.2% Triton X on ice for 15 min to activate the antigen. Blocking was accomplished using a Nichirei Histofine SAB-PO (R) kit (Tokyo, Japan). The sections were treated with 10 mg/mL of AGE polyclonal antibody (bs-1158R. Bioss, Boston, MA) in PBS containing 2% BSA and incubated overnight at 4 °C. Streptavidin–biotin-peroxidase and 3,3′-diaminobenzidine tetrahydrochloride were used to visualize immunoreactivity using the Histofine SAB-PO (R) kit (Nichirei, Tokyo, Japan).

### Ex Vivo adhesion of *M. nonliquefaciens* to the cornea of mice

Diabetic mice, diabetic mice that consumed pyridoxamine, age-matched wild-type mice, and young wild-type mice were used, with five mice from each group. Six-fold diluted PA-IODO ophthalmic and eye-washing solution (Nihon Tengan Kenkyujyo, Aichi, Japan) and PBS were used to rinse the excised corneas. The corneas were placed in 1 mL of SHEM containing 30 μL of the bacterial solution and incubated for 1 h at 37 °C. The bacterial solution was adjusted to 1.0 × 10^8^ CFU/mL using the calibration curve as that in in vitro experiments. Following PBS rinsing, the corneas were crushed twice using the Micro Smash MS-100 system (Tomy Seiko, Tokyo, Japan) at 825* g* for 1 min. The crushed corneas were diluted in PBS and applied to the sheep blood agar. The agar was incubated for 24 h at 37 °C in a humid environment with 5% CO_2_, and the number of colonies on the agar was counted manually. We performed this experiment three times.

### Statistical analysis

Each experiment was repeated three times. The representative data are shown in the figures. The data were analyzed using JMP statistical software, version 11.2.0 (SAS Institute, Cary, NC), using a two-tailed unpaired t-test. *P*-values < 0.05 were used to denote statistical significance.

## Data Availability

The datasets analyzed during the current study are available from the corresponding author (HI) on reasonable requests.
